# Should Israel screen all mothers-to-be to prevent early-onset of neonatal group B streptococcal disease? A cost-utility analysis

**DOI:** 10.1186/2045-4015-2-6

**Published:** 2013-02-20

**Authors:** Gary M Ginsberg, Arthur I Eidelman, Eric Shinwell, Emilia Anis, Reuven Peyser, Yoram Lotan

**Affiliations:** 1 Medical Technology Assessment Sector, Ministry of Health, Jerusalem, Israel; 2 Department of Neonatology, Shaare Zedek Medical Center, Jerusalem, Israel; 3 Department of Neonatology, Kaplan Hospital, Rehovot, Israel; 4 Division of Epidemiology, Ministry of Health and Braun School of Public Health, Hebrew University and Hadassah, Jerusalem, Israel; 5 Sackler School of Medicine, Tel-Aviv University, Tel-Aviv, Israel; 6 Ambulatory Services Division, Ministry of Health, Jerusalem, Israel

**Keywords:** Cost utility analysis, Group B streptococcal disease, Screening, Intrapartum antiobiotic prophyhlaxis

## Abstract

**Background:**

In Israel, an average of 37 children are born each year with sepsis and another four with meningitis as a result of Group B Streptococcal (GBS) disease. Israel currently only screens mothers with defined risk factors (around 15% of all pregnancies) in order to identify candidates for Intrapartum Antiobiotic Prophyhlaxis (IAP) of GBS. This paper presents a cost-utility analysis of implementing an alternative strategy, which would expand the current protocol to one aiming to screen all pregnant women at 35–37 weeks gestation based on taking a vaginal culture for GBS.

**Methods:**

A spreadsheet model was built incorporating technical, epidemiological, health service costs, demographic and economic data based primarily on Israeli sources.

**Results:**

The intervention of universal screening (compared with the current scenario) would increase screening costs from 580,000 NIS to 3,278,000 million NIS. In addition, the intervention would also increase penicillin costs from 39,000 NIS to 221,000 NIS. Current culture screening of approximately 15% of mothers-to-be with high risk factors resulted in 42 GBS births in 2008-9 (0.253/1000 births). Expanding culture screening to 85% of mothers-to-be, will decrease the number of GBS births to 17.3 (0.104/1000 births). The initial 2.9 million NIS incremental intervention costs are offset by decreased treatment costs of 1.9 million NIS and work productivity gains of 811,000 NIS as a result of a decrease in neurological sequelae from GBS caused meningitis. Thus the resultant net cost of the intervention is only around 134,000 NIS. Culture based screening will reduce the burden of disease by 12.6 discounted Quality Adjusted Life Years (QALYS), giving a very cost effective baseline incremental cost per QALY (cf. risk factor screening) of 10,641 NIS per QALY. The data was very sensitive to rates of anaphylactic shock and changes in the percentage of meningitis cases that had associated long term-sequelae.

**Conclusion:**

It is recommended that Israel adopt universal culture-based GBS screening.

## Background

Group B streptococcal (GBS) disease is a significant cause of neonatal sepsis and early neonatal mortality within the first week of life [early onset neonatal (EON) diseases]. Intra-partum antibiotic prophylaxis (IAP) has been documented to lower the incidence of EON GBS disease. The current practice in Israel is to perform culture screenings for GBS in mothers according to the presence of risk factors (e.g. pre-labor membrane rupture, preterm labor, intra-partum fever). Women with identified risk factors are then treated with antibiotics (IAP).

Consideration is now being given to implement a program for universal screening of pregnant women in Israel using a vaginal-anal culture taken at 35–37 weeks of gestation. Mothers-to-be found with a positive culture test would then receive IAP. Our model assumes that the culture will be taken by a physician/nurse at 35–37 weeks gestation. This timing is current practice and avoids the possibility of lower positive predictive values that might occur in self-administered cultures or cultures taken taken before 35 weeks gestation.

In order to aid the decision-makers in their choice as to whether or not to adopt this new intervention strategy, this study will carry out a cost-utility analysis based on calculating the cost per Quality Adjusted Life Year (QALY) of implementing a universal screening strategy for GBS in Israel.

## Methods

GBS colonization rates of 14.96% were based on weighted ethnic specific colonization rates (n= 1,329) from two recent Israeli studies [1,2]. Based on data from the Israel Center for Disease Control and the Ministry of Health, the incidence rate of early onset GBS in 2005-2006 in Israel was 0.315 per 1000 births and 0.253 per 1000 births in 2008-9.

Assuming sensitivity and specificities for culture screening of 0.95 and 0.97 respectively [3], we back-calculated, the underlying natural incidence (null scenario) of early onset GBS, under the baseline assumption in 2008-2009, that 15% of the mothers-to-be nationwide received culture screening on an opportunistic basis (see Appendix 1 for full details).

Finally we estimated the incidence in a future intervention scenario where 85% of mothers-to-be would receive culture screening, a figure currently attained in the USA (3). Of the 15% who will not be screened, around half were unscreened due to pre-term births [4].

We assumed that mothers who were given a scheduled C-section birth would not receive IAP. Currently 18.9% of Israeli births [4,5] are delivered by C-section of which 30% are scheduled in advance and so would not receive IAP.

Based on evidence from a Cochrane meta-analysis [6], we assumed that IAP with penicillin would prevent 83% of the true positive identified cases from giving birth to a child infected with GBS. We assumed that 0.022% of the mothers who received IAP would develop an anaphylactic reaction [7], with an attendant case fatality rate (CFR) of 10% [8].

In 2010, there were 166,184 live births (including 3,784 twins and 77 other multiple births) and 501 still births, resulting from 162,740 deliveries in Israel [9]. By multiplying the birth rate by the GBS incidence rate and adjusting for the efficacy of the intervention, we calculated the number of infants born with GBS in the following three scenarios: the null scenario, the present scenario and the future intervention scenario, where 85% of mothers to be would be culture-screened at 35–37 weeks.

Ninety percent of the children with EON GBS, were assumed to suffer from only sepsis, whilst the remaining 10% had meningitis in addition to the sepsis, based on the past ten years experience of the Shaare Zedek Hospital in Jerusalem.

The reported range of the percentage of meningitis cases that develop long term neurological sequelae increased from 28.4% (n=218) in studies from 1973-1985 [10-17] to 47.5% (n=141) in studies reported from 2000-2012 [18,19]. As no studies were identified during the period 1986-1999, the baseline estimates used in our model were based on the 47.5% figure from the two recent studies from the 21^st^ century [18,19]. In addition, we conducted a sensitivity analysis over a range of values suggested by the literature.

The neurological sequelae consisted of severe (31.3% of all neurological sequellae), moderate (33.4%) and mild (35.3%) neurological disorders [18,19]. In addition a further 4.6% of meningitis cases suffered from severe or profound bilateral hearing loss [13].

### Costs of intervention

No additional marginal costs were imputed for the taking of the culture as this was considered to be part of the routine examination of the mother-to-be. Laboratory costs of analyzing the culture test (including the culture tube) were 25.62 NIS per test. No provision was made for physician labour time as this was considered to be part of the routine pre-natal check-up in Israel.

Penicillin costs were based on an average of two doses (given four hours apart) per birth (average delivery time 6–8 hours) of 5 million units of benzyl penicillin costing 11 NIS per 10mu vial (Personal Communication: Alan Greenberg, Chief Pharmacist, Shaare Zedek Hospital, Jerusalem).

No extra marginal cost was incurred for nurses time in setting up the IV drip.

### Costs of anaphylactic shock, GBS cases and sequelae

Each fatal anaphylactic shock case was assumed to cost one day's stay in an Intensive Care Unit (5,142 NIS). Non-Fatal anaphylactic cases were assumed to spend an extra two days in hospital [20] costing 4,208 NIS.

Hospital costs (in relation to non-GBS births) of 29.458 NIS and 37,872 NIS for GBS sepsis and sepsis with meningitis cases were based on 14 and 18 days stay respectively (obtained from an analysis of the case-notes of 24 infants hospitalized for GBS in Shaare Zedek Hospital, Jerusalem) in a quasi- Intensive Care Unit (ICU) at 2,106 NIS per day (Ministry of Health, Price List 2010). Fatal cases were assumed to cost the equivalent of one days stay (5,142 NIS) in an ICU.

Post hospital care (e.g. outpatient follow up visits) was assumed to amount to 36.0% of in-hospital care costs [21]. Both in- and post-hospital costs for GBS children were adjusted by factors of 38.8% and 21.0% respectively in order to estimate the additional costs for GBS children compared with births to children without GBS [21].

Costs of acute care were calculated by multiplying the expected number of cases by the unit costs of health services received. For chronic sequelae (deafness, and brain damage), the expected age-specific number of cases was multiplied by the age-specific discounted lifetime costs.

Costs of treating long-term sequelae were based on lifetime costs discounted at 3% per annum. Data on costs of deafness were retrieved from a recent cost-utility analyis of cochlear inplants^a^. These costs included cost of aids or implants, hearing tests, ear moulds, special therapy, school visits, acoustic classrooms, amplification for teachers, remedial education for children with and without cognitive complications.

Lifetime costs were estimated to be around 2,640,519 NIS, 1,281,790 NIS and 316,180 NIS respectively for severe, moderate and mild neurological brain damage^b^. These costs included initial diagnostics and care costs, care costs in subsequent years, additional medical costs and special education costs.

Age-specific lifetime costs of caring for brain damage (diagnosis, medical care, special education, rehabilitative day care, residential care) were obtained from the ministry of health^a,b^[22] and the ministry of social affairs^c^.

Around 75% and 5% of severe and moderate-mild brain damaged persons were assumed to be cared for in residential care centers, reflecting the trend of de-institutionalisation [23] at a cost of around 105,000 NIS per year.

Since the analysis was from a social perspective, estimates of lost productivity due to sequelae were included and based on the assumption that persons with moderate or severe sequelae did not participate in the workforce. Hearing challenged persons and persons with mild sequelae were assumed to participate fully in the labor force. Average discounted lifetime employment costs of 1,102,287 NIS per person were calculated from national average gross wages [9] increased by a factor of 15% to include employers pension, national insurance and educational fund contributions and by age-specific unemployment rates [9].

### QALYS gained

QALY losses due to morbidity of GBS caused sepsis and sepsis-meningitis cases were calculated by multiplying the expected number of cases by the expected duration of the illness (14 days and 18 days respectively), and the disability weights of 0.31 [24] and 0.62 [25] respectively attached to acute illness and the age-specific average QALY weight of an infant not affected by GBS sepsis or meningitis^a^.

QALY losses due to morbidity from chronic sequelae from meningitis were calculated by multiplying the expected number of sequelae by the average lifelong disability weight of 0.964 [26-28] attached to the sequelae and the age-specific average QALY weight of a person not affected by GBS meningitis [25]. The results were discounted at a rate of 3% per annum over the persons remaining life expectancy [9].

QALY losses due to morbidity from anaphylactic reactions were based on an assumed disability weight for meningitis (0.61) and the average duration of acute reaction was assumed to be one day.

QALY losses due to mortality were estimated from the product of the following:

– the number of incident cases

– case-fatality rate of 2.8% representing the excess mortality in GBS as opposed to non-GBS cases [29,30] or the 10% case fatality rate from anaphalactic reactions [8].

– age and gender specific life expectancies at birth of 80.2 for males and 82.1 for females [9] in Israel,

– age-specific QALY weights of a healthy person^a^.

### Cost-utility analysis

A spreadsheet model was built incorporating technical, epidemiological, health service utilization and costs, demographic and economic data described above.

The cost utility ratio calculated the net costs per Quality Adjusted Life Year (QALY) added of the intervention of universal screening followed by IAP prophylactics, using the formula.

$$\mathrm{Net}\;\mathrm{Costs}\;\mathrm{per}\;\mathrm{QALY}\;=\frac{\text{Costs}\;\mathrm{of}\;\text{intervention}–\mathrm{Savings}\;\text{in}\;\mathrm{treating}\;\text{GBS}}{\text{QALYs}\;\mathrm{added}\;\mathrm{from}\;\text{averted}\;\text{mortality}\;\text{and}\;\text{morbidity}}$$

All costs are at presented in mid-2010 price levels, at the average annual exchange rate of 3.588 shekels to the US dollar [9]. Costs are viewed from a societal perspective (i.e. including estimates of lost productivity in addition to health and welfare services costs).

Estimates of QALYs added by the intervention do not include those arising from reduced caregiver burden for sequelae since such data is not available. All future costs and QALYS were discounted at an annual rate of 3%.

Taking into account the resources available in Israel, an intervention is defined as being very cost-effective and cost-effective if the cost per QALY is less than the per capita GNP of 106,548 NIS in 2010 [9] or between 1–3 times the per capita GNP (106,548-319,644 NIS) respectively. If the cost per QALY is more than three times the GNP per capita (319,644 NIS) then the intervention is regarded as not being cost-effective [31].

Averted QALY losses are calculated by summing the mortality and morbidity gains from decreased incidence of GBS as a result of the universal screening intervention.

## Results

The intervention of universal screening (compared with the current scenario) would increase screening costs from 578,000 NIS to 3,278,000 million NIS. In addition, the intervention would also increase penicillin costs from 39,000 NIS to 221,000 NIS. The gross annual cost of the intervention is therefore 3,499,000 million NIS compared with 617,000 NIS under the current scenario, an increase of 2,881,000 NIS.

The estimated natural incidence in Israel (i.e. in the null-scenario of absence of any culture screening) is 0.30 per 1000 births resulting in 49.8 GBS births. As a result of the estimated increase in culture screening to around 15% in 2008-9, there was a decrease in GBS incidence to 0.2525 per 1000 births in 2008-2009 (42.0 GBS births). With the expansion of culture screening 85% of mothers to be, the incidence rate will fall to 0.10 per 1000 births (17.3 GBS births).

There will be around 24.7 fewer GBS births as a result of universal screening. Treatment costs will decrease by 1,936,000 NIS (Table 1), of which 83.5% (1,620,000 NIS) is attributable to decreased costs of long-term sequelae. In addition, there will be gains of 812,000 NIS in work productivity as a result of a decrease in medium and severe neurological cases from 1.25 to 0.52 persons, resulting in the intervention saving a total of 2,747,000 NIS (after rounding).

**Table 1:** Savings (vs. null scenario) as a result of implementing universal screening for GBS (NIS at 2010 price levels)

**TREATMENT COSTS**	**Marginal**	**Risk-factor**	**Universal**	**Savings**
	**Unit costs**	**Screening**	**Screening**	
Hospitalization				
Fatal and non-fatal anaphylaxis	4,301	3,686	20,890	
Fatality vs. non-GBS	1,997	2,347	968	
Sepsis vs. non GBS	11,441	419,988	173,336	
Meningitis & sepsis vs. non-GBS	14,710	59,998	24,762	
Post-Hospital Care vs. non-GBS	2,130	89,393	36,894	
		575,412	256,851	*318,561*
LTC				
Deafness	508,260	104,858	43,227	
Severe Neuro	2,640,519	1,604,008	662,002	
Moderate Neuro	1,281,790	828,425	341,906	
Mild Neuro	316,180	216,369	89,299	
		2,753,659	908,003	*1,617,175*
Total Health Service Costs		2,775,870	1,136,483	*1,935,737*
Lifetime productivity losses		1,382,007	570,378	*811,828*
Total Societal Costs		4,711,077	1,963,713	*2,747,365*

On account of the savings in treatment and care costs and the productivity gains due to the decrease in the number of GBS sequelae, the marginal incremental costs of Universal GBS screening falls from 2,881,000 NIS to only 134,000 NIS (Table 2).

**Table 2:** Summary of costs (NIS at 2010 price levels) and QALY losses of proposed universal GBS screening versus current risk-factor screening

			**Incremental**
	**Current**	**Proposed**	**Change**
**Screening Costs**	617,408	3,498,644	2,881,236
**Treatment Savings**	–614,683	–2,550,420	–1,935,737
**lifetime productivity gains**	–256,990	–1,068,618	–811,628
**Net Cost to Society**	–254,266	–120,394	133,872
**Mortality QALYs lost**	34.97	23.87	–11.10
**Morbidity QALYs lost**	0.56	0.24	–0.32
**Sequellae QALYs lost**	1.97	0.81	–1.16
**Total QALYS lost**	37.50	24.92	–12.58

In the current scenario, 37.5 QALYS are lost annually due to GBS. Adopting universal screening will reduce this burden to 24.9 QALYS, a gain of 12.6 (discounted) QALYS (Table 2). Therefore, the baseline incremental cost per QALY (cf the current scenario) is a very cost-effective 10,641 NIS (133,872/12.58).

Net costs to society and health services per case of GBS prevented are 5,432 NIS and 38,367 NIS, while net costs per neurological sequelae case prevented is 117,616 NIS and 830,696 respectively.

### Sensitivity analyses

Our calculations assumed sensitivity and specificity of culture screening that were based on the use of an enrichment broth that improves detection [3], which is currently routinely used in Israel. Using a lower sensitivity rate of 0.87 and specificity of 0.96 [32] will increase the cost (373,482 NIS) per QALY (10.85) ratio to 34,426 NIS per QALY.

Due to the linear structure of the underlying model, the data was totally insensitive to changes in the current percentage of persons being screened using cultures. The same value of 10,641 NIS per QALY applied even when the assumed culture screening rate in 2008-9 was only 10% or if it was 23% (i.e. the Maccabi health services data applied to the whole population).

However the data was sensitive to changes in the percentage of meningitis cases that had long term-sequelae as well as the incidence rate of anaphylactic shock (Table 3). If the meningitis sequelae rate were to be 22.5%, then costs per QALY would rise to 117,993. NIS, rendering the intervention to being cost-effective. If the rate of anaphylactic shock rate were to be 0.001% [8,33] or zero, the cost per QALY ratio will fall to 5,749 or 5,558 respectively. The data was also sensitive to changes in GBS carrier prevalence rates as shown in relation to meningitis sequelae rates in Table 4.

**Table 3:** Cost per QALY from societal perspective

**% getting**	**% of meningitis cases with long-term sequelae**			
Anaphalaxis From IAP	**22.5%**	**35%**	47.5%*	**60%**
**0%**	68,536	36,609	5,558	cs
**0.001%**	69,883	37,336	5,749	cs
**0.004%**	74,252	39,690	6,155	cs
0.022%*	117,993	63,025	10,641	cs
**0.040%**	279,253	145,536	25,857	cs

* baseline rate.

cs denotes cost-saving.

**Table 4:** Cost per QALY from societal perspective

**GBS carrier**	**% of meningitis cases with long-term sequelae**			
Prevalence	**22.5%**	**35%**	**47.5%**	**60%**
**0.2000**	222,459	152,325	86,209	24,031
0.2525*	117,993	63,072	10,641	cs
**0.3000**	71,705	23,138	cs	cs

* baseline rate.

cs denotes cost-saving.

## Discussion

The estimated cost per QALY of 10,641 NIS is very-cost effective according to the WHO criteria putting it on a par with other various public health, preventive and curative interventions in Israel. Investment of an additional 2.9 million NIS of resources will in effect only cost around 134,000 million NIS in the long-term. Even using less sensitive direct agar plating [3], resulted in a cost per QALY ratio that was still very cost-effective, costing less than 32% of GNP per head.

Our finding that the marginal impact of expanding the culture screening intervention (from 10% to 85%) was very cost-effective was in keeping with results found with different models in other countries. A UK study found various culture testing interventions to be cost-saving relative to a no intervention strategy [34]. A Dutch study [35] found a combined screening and risk-based study to be very cost-effective, while for screening alone the cost per QALY was around twice the GNP per head, rendering it just cost-effective. In addition, a recent USA article suggests that universal treatment of term pregnancies with a prior history of GBS colonization is more cost-effective than a strategy of screening and treating based on positive culture results [36].

Our model used a compliance rate of 85% based on the US experience [3] and the fact that Israel has a well-developed and efficient public health service which reaches a much higher proportion of society than in countries such as the US. In addition in the US many women do not have full health insurance coverage as opposed to Israel where there is universal health coverage. In any case, if a lower (or even higher) compliance rate is achieved, this will not alter the cost-utility ratios as both the cost numerator and QALY denominator will be affected in the same proportions.

The use of intrapartum Polymerase Chain Reaction (PCR) and optical immunoassay (OIA) screening methods were not investigated in this paper because the relatively high costs of these tests have resulted in them being totally dominated in a recent British Cost-Utility analysis [37] by the cheaper and more effective culture test followed by IAP intervention scenario. However despite its current high cost per QALY, PCR could in the future offer benefits when used in the delivery room. While this could provide a useful complementary adjunct to universal screening, it does not offer the potential to be a substitute for culture-based screening.

Costs per QALY for IAP are overestimated to the extent that

we were unable to estimate transport costs to receive treatment and out-of-pocket expenses for sequelae.

we did not include any long-term sequelae from sepsis cases without meningitis [38]

we did not include the marginal savings in physicians time (approximately 7 NIS per mother-to-be) in order to identify and record risk factors that would indicate GBS screening is required, since use of these factors will not be required.

The colonization rate used in our model was 14.96% [1,2]. This is of similar magnitude as rates reported each year by the Shaare Zedek Medical Center in Jerusalem and the rate of 16.9% based on the results of over 60,000 screening cultures taken by Maccabi Health Services (the second largest HMO in Israel) from 2004-2011. [39-43]. The Maccabi data was not used as the membership of this Health Service tends to have a higher socio-economic profile than the country as a whole.

Our results were very sensitive to the percentage of meningitis cases that have long-term sequelae. However even the incidence of long-term sequelae would have to fall below 25.05% to downgrade the intervention from being very cost-effective to just cost-effective. If however, long-term sequelae rates were to rise above 50.1% then the intervention becomes cost-saving as the costs averted of treating the sequelae outweigh the intervention costs.

If the severity mix of sequelae was less severe (e.g. the ratio of severe to mild cases decreases), then due to the large differences in lifetime costs per case, the costs per QALY of the intervention would rise.

Our analysis assumed that all mothers who were culture positive would be given IPA in Israel where there is universal national health insurance and good accessibility to both pre-natal and curative health services. Current experience in Israel points to 100% compliance for those mothers-to-be who are currently culture tested. This 100% compliance rate is also used in analyses carried out in the UK National Health Services [44] and compares favorably with the USA where due to different health system structures, only around 80% of mothers with an indication for IAP actually received it appropriately [44].

Opponents to the introduction of universal screening for GBS bring up the specter of increased development of drug resistant GBS strains as well as other bacteria that will become resistant to penicillin. However, in actuality according to CDC guidelines, the advent of universal screening in the states has NOT been associated with increased resistant organisms [45].

In the US, under universal screening a greater than expected number of cases of early onset GBS occurred among infants born to women with negative prenatal screening results. This phenomena of false negatives may have been caused by a variety of local factors including the culturing technique, the use of recommended transport medium, and the laboratory culture protocol [46] and we believe would be less likely to occur in Israel due to its universal health coverage and more extensive public health network of ante-natal clinics.

## Conclusion

In summary, on the basis of the cost-utility analysis, that integrates epidemiological, clinical and economic data, it is recommended that Israel adopt universal GBS screening.

## Endnotes

^a^Ginsberg G.M, Stein-Zamir C. Preliminary Cost-Utility analysis of national infant vaccination against Meningococcal Serogroup B disease in Israel. Ministry of Health. Article in Process.

Ginsberg G.M. Cost-Utility Analysis of Unilateral and Bilateral Cochlear Implants in Israel. Internal Document, Medical Technology Assessment, Ministry of Health, 8th March 2008.

Ginsberg G. Guidelines for the submission of Cost Utility Analyses of candidate interventions for the Health Basket, Israel Ministry of Health (2009).

^b^Personal Communication: Prof. Asher Or-Noi, Director of Child Development and Rehabilitation, Ministry of Health.

^c^Personal Communication: Prof. Joav Merrick, Office of the Medical Director, Health Services, Division for Mental Retardation, Ministry of Social Affairs.

## Appendix I: estimation of natural and projected GBS rates

The natural incidence rate of GBS (R0) is the rate that would have occurred in the absence of any screening program or IAP usage is calculated using the following formulae:

$$\mathrm{RND}15=R15/\left(100\%-C\%\right)$$

$$\begin{array}{l} R0=\mathrm{RND}15+\mathrm{RND}15\;\times \;\left(S15-S0\right)\;\times \;\mathrm{SEN}\\ \;\times \;\mathrm{EFF} \end{array}$$

Similarly, the expected rate of GBS (R85) if 85% of mothers-to-be are screened in a nationwide program will be:

$$\begin{array}{l} R85=\mathrm{RND}15+\mathrm{RND}15\;\times \;\left(S85-\text{S}15\right)\;\times \;\text{SEN}\\ \;\times \;\text{EFF} \end{array}$$

Where

SEN = Sensitivity of culture screening to discover GBS.

EFF = Efficacy of penicillin to prevent GBS.

R85 = Rate of GBS in all deliveries with culture screening of 85% of mothers-to-be.

S85 = Screening rate of 85%

RND15 =Rate of GBS in all deliveries (excluding C-sections known in advance) with culture screening of 15% of mothers-to-be.

R15 = Rate of GBS in all deliveries with culture screening of 15% of mothers-to-be.

S15 = Screening rate of 15%

R0 = Rate of GBS in all deliveries with no culture screening programme.

S0 = Screening rate of 0%

C = percentage of C-section births known in advance

## Abbreviations

EON: Early onset neonatal; GBS: Group B streptococcal; IAP: Intrapartum antiobiotic prophyhlaxis; ICU: Intensive care unit; QALY: Quality Adjusted Life Year.

## Competing interests

The authors declare that they have no competing interests.

## Authors’ contributions

GG collected the data, built the model, co-ordinated the study and drafted the manuscript. AE participated in the design of the study, provided medical know-how, carried out a utilization study and drafted the manuscript. ES, EA & RP provided technical knowledge and data for the study. YL conceived of the study, and participated in its coordination and helped to draft the manuscript. All authors read and approved the final manuscript.

The opinions expressed in this paper are those of the authors alone and not of the institutions with which they are affiliated.
